# Management of pregnancy in patients with Graves’ disease secondary to alemtuzumab: a case series and literature review

**DOI:** 10.3389/fendo.2026.1955471

**Published:** 2026-09-28

**Authors:** Federico Crapella, Ilaria Muller

**Affiliations:** 1 Department of Clinical Sciences and Community Health, Department of Excellence 2023-2027, University of Milan, Milan, Italy; 2 Endocrinology Unit, Fondazione IRCCS Ca’ Granda Ospedale Maggiore Policlinico, Milan, Italy

**Keywords:** alemtuzumab (Lemtrada), gestation, Graves’, hyperthyroidism, hypothyroidism, pregnancy, TSH receptor antibodies (TRAb)

## Abstract

**Introduction:**

Alemtuzumab treatment for multiple sclerosis frequently induces Graves’ disease (GD), characterized by common coexistence of stimulating and blocking anti-thyrotropin receptor autoantibodies (TRAb), causing fluctuations of thyroid function. Alemtuzumab-induced TRAb-positive hypothyroidism is also common. These conditions are challenging to manage during pregnancy, considering the imperative necessity to maintain euthyroidism to minimize risks for both pregnancy and fetal/neonatal outcomes.

**Methods:**

We reviewed the clinical course and therapeutic management of four pregnant women affected with alemtuzumab-induced GD or TRAb-positive hypothyroidism followed at our Institution, and seven additional cases published in literature.

**Results:**

At our institution two patients with alemtuzumab-induced GD (one diagnosed before pregnancy, the other during the third trimester) showed a good control of thyroid function on anti-thyroid drugs (ATD). Two women had alemtuzumab-induced TRAb positive hypothyroidism; during pregnancy thyroid function was partially controlled with levothyroxine (LT4) alone in one case, and fully controlled with block and replace (ATD and LT4) in the other case. No pregnancy or fetal/neonatal negative outcomes were observed, except for preterm delivery of a healthy baby in Case 3, due to emergency cesarean section for pre-eclampsia progressing to HELLP syndrome. The literature search showed seven additional cases of pregnant women with alemtuzumab-induced GD (N = 5) or TRAb-positive hypothyroidism (N = 2), mainly diagnosed during gestation, especially in the first trimester. Six newborns developed transient thyroid dysfunction, often requiring treatment and later resolved.

**Conclusion:**

The clinical management of alemtuzumab-induced thyroid dysfunction during pregnancy is challenged by frequent difficult maternal biochemical thyroid control and TRAb-mediated fetal/neonatal thyroid dysfunction. These patients need to be managed in a specialist setting, with close monitoring of maternal thyroid function and fetal/neonatal parameters, especially in cases with high TRAb titers.

## Introduction

Alemtuzumab is a humanized monoclonal antibody that binds to the CD52 antigen and causes rapid depletion of B and T lymphocytes, followed by a phase of immune reconstitution characterized by differential repopulation of lymphocyte subsets (1, 2). Since 2013, alemtuzumab has been approved for the treatment of highly active relapsing-remitting multiple sclerosis (MS) in adults, demonstrating efficacy in reducing relapse frequency and disability progression (3, 4). Despite its efficacy in treating MS, alemtuzumab is associated with frequent autoimmune complications as adverse effects of immune reconstitution (5, 6). Thyroid autoimmunity is common, with a reported incidence between 30% and 40% of treated patients, and occurring several years after treatment, with a peak 2 to 3 years after the first dose (5, 7, 8). Alemtuzumab-induced Graves’ disease (GD) is the most frequent manifestation (up to 65% of cases) and presents distinctive clinical and immunological features compared to the classical form, including a frequently fluctuating course of thyroid function between hyperthyroidism and hypothyroidism, due to the coexistence of autoantibodies to the thyrotropin receptor (TRAb) with both stimulating and blocking function (6, 9, 10). TRAb-positive hypothyroidism is also a frequent complication, occurring in 21-34% of total cases of alemtuzumab-induced thyroid dysfunction, and mainly due to the presence of blocking TRAb (11).

During pregnancy, the management of GD is particularly complex, as it is necessary to maintain adequate thyroid hormone concentrations for both mother and fetus, minimizing the risk of maternal, fetal, obstetric and neonatal complications (12, 13). Furthermore, TRAb cross the placenta and can stimulate the fetal thyroid gland, resulting in fetal and neonatal hyperthyroidism when maternal TRAb titers are significantly elevated (14, 15).

## Methods

We identified four cases of pregnant women affected with alemtuzumab-induced GD or TRAb-positive hypothyroidism among the cohort of patients followed in the dedicated clinic at our Institution: Department of Endocrinology, Fondazione IRCCS Ca’ Granda Ospedale Maggiore Policlinico di Milano, Italy. All patients provided informed consent for publication of this case series.

In one case alemtuzumab-induced thyroid dysfunction developed during pregnancy, while in three cases it was diagnosed before conception. We collected data about endocrine and neurological clinical history, previous MS treatments, course of thyroid function and serum autoantibodies titers, therapeutic management and fetal/neonatal health status. In particular, the evaluated thyroid-related serum laboratory parameters included thyroid stimulating hormone (TSH), free thyroxine (FT4), free tri-iodothyronine (FT3), TRAb, anti-thyroglobulin autoantibodies (TgAb), and anti-thyroperoxidase autoantibodies (TPOAb). We don’t report the assay methods since they were measured in different laboratories during the years of monitoring requested in these patients. The concentration of stimulating and blocking TRAb was performed in one case using in-house luciferase bioassays as previously described. Briefly, Chinese Hamster Ovary (CHO) cell lines stably transfected with the human TSH-receptor and a cAMP responsive luciferase reporter were incubated with human sera; stimulating TRAb were considered present with stimulation index (SI) values >1.5, while blocking TRAb were considered positive if the inhibition index (InI) was >20% (16). We also conducted a literature search in the PubMed, Embase and Google Scholar online databases until April 2026, using the following search keywords: (“Graves” OR “hyperthyroidism” OR “hypothyroidism” OR “thyroid dysfunction” OR “thyroid”) AND (“alemtuzumab” OR “immune reconstitution therapy”) AND (“pregnancy” OR “gestational”). We searched for published case reports/series of pregnant women with alemtuzumab-induced thyroid dysfunction providing details of their clinical management during gestation, and fetal/newborn outcome when available. After providing full-text assessment of potentially eligible reports, we included seven additional cases; no duplicates were identified.

## Results

**Figure 1** summarizes the main thyroid alterations observed in the four patients described in this case series, from the beginning of pregnancy to the immediate postpartum period.

**Figure 1:**
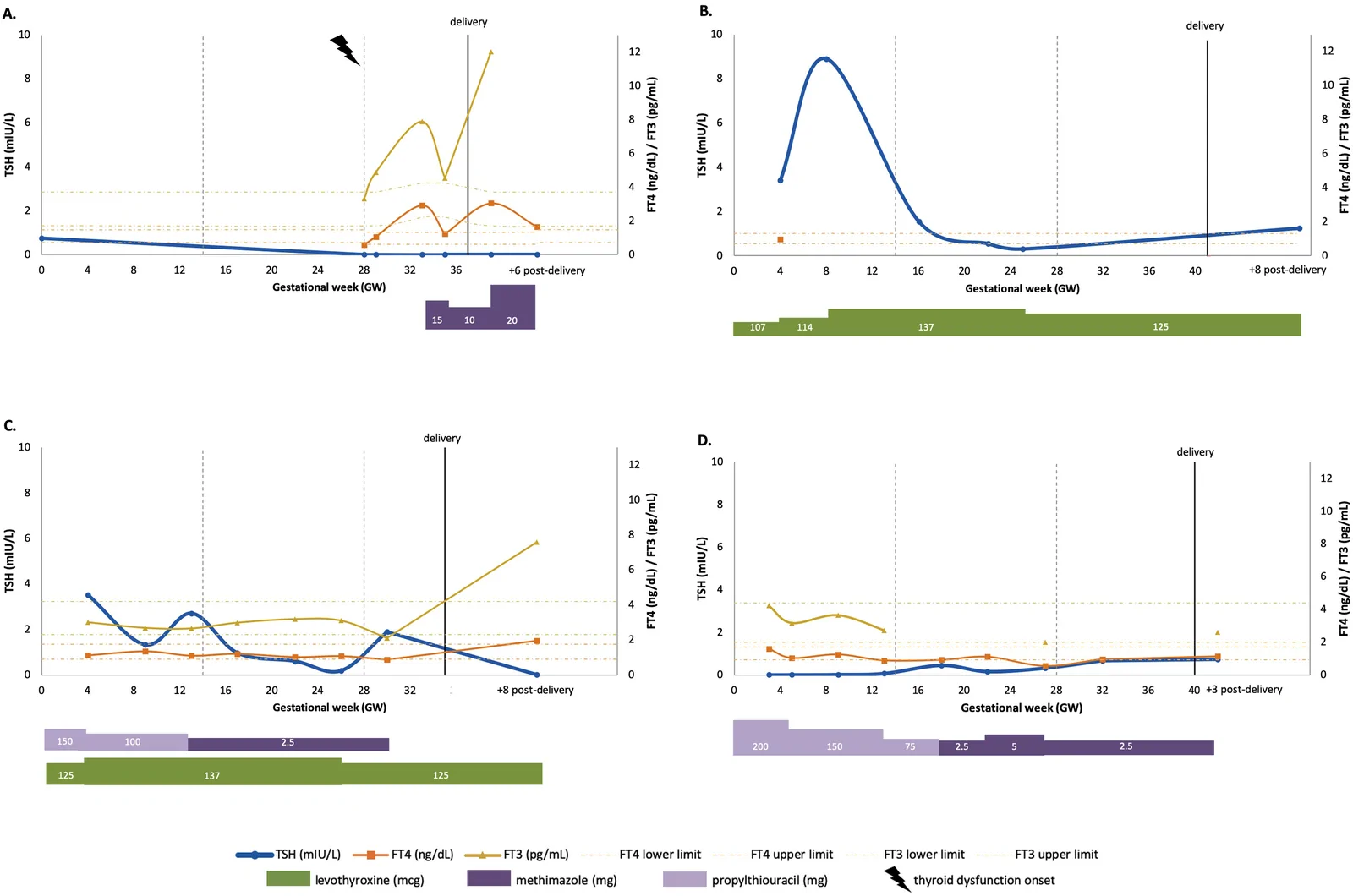
Thyroid function and treatment during pregnancy and in the immediate postpartum period in the four patients with alemtuzumab-induced thyroid dysfunction followed at our Institution. Trends of thyroid-stimulating hormone (TSH), free-thyroxine (FT4) and free tri-iodothyronine (FT3) serum concentrations alongside their respective reference intervals, and adjustments in maternal treatments, displayed according to the gestational weeks (GW). **(A)**, Case 1; **(B)**, Case 2; **(C)**, Case 3; and **(D)**, Case 4.

### Case 1

A 19-year-old woman was diagnosed with MS and treated with steroids, interferon and fingolimod (a sphingosine 1-phosphate receptor modulator that sequesters lymphocytes in primary lymphoid organs) (17). During the following years she had several disease relapses and developed optic neuritis, thus after 8 years from the initial MS diagnosis she was treated with two doses of alemtuzumab one year apart. Before such therapy, she was euthyroid with negative serum thyroid autoantibodies, except for slightly positive TgAb (97 IU/mL; negative if <4), and she had normal thyroid ultrasound and scintigraphy scans.

At age 29, two years after the first alemtuzumab infusion, she became pregnant for the first time. Her thyroid function was normal (TSH 0.75 mIU/L) with negative TgAb and TPOAb. At gestational week (GW) 28 repeated thyroid function tests showed subclinical hyperthyroidism due to GD: TSH 0.01 mIU/L, TRAb 9.2 IU/L (negative if <1.8) and positive TgAb and TPOAb. She had normal FT3 (3.31 pg/mL; normal reference range [nrr] 1.71-3.70) with low FT4 (0.59 ng/dL; nrr 0.70-1.48) serum concentrations, likely reflecting hypothyroxinemia during pregnancy. Her thyroid ultrasound showed a normal size and morphology, with slightly hypoechoic texture. At GW 33 she progressed to overt hyperthyroidism with TSH<0.001 mIU/L, FT4 2.91 ng/dL (nrr 0.60-1.34), FT3 7.88 pg/mL (nrr 2.21-4.23), and was started on methimazole (MMI) 15 mg daily for three weeks, and then 10 mg daily, with marked improvement of her thyroid function: TSH <0.001 mIU/L, FT4 1.23 ng/dL (nrr 0.60-1.34), FT3 4.53 pg/mL (nrr 2.21-4.23), TRAb 3.8 IU/L (negative if <1.8). Fetal parameters were normal at ultrasound; the delivery occurred by scheduled caesarean section at GW 37 and the newborn was healthy.

In the first six weeks postpartum her hyperthyroidism worsened and required MMI dose increase up to 30 mg daily: TSH <0.001 mIU/L, FT4 3.04 ng/dL (nrr 0.70-1.48), FT3 11.99 pg/ml (nrr 1.70-3.70), with TRAb constantly positive (6.5 IU/L, negative if <1.8). From the fifth postpartum month her thyroid control improved, TRAb became negative and MMI was progressively tapered to a maintenance dose of 1.25 mg daily and finally discontinued two years after delivery.

About three years after the first maternity, she started another pregnancy. In the first trimester her thyroid function tests showed abnormalities most likely related to hyperemesis gravidarum, for which anti-thyroid drugs (ATD) were not restarted: TSH 0.07 mIU/L, FT4 1.07 ng/dL (nrr 0.60-1.34), FT3 3.64 pg/mL (nrr 2.20-4.20), TRAb <0.26 IU/L (negative if <1.8). Thyroid function then normalized spontaneously and remained stable throughout the pregnancy, which ended again with caesarean section. Six months after the second delivery she had an episode of post-partum thyroiditis, then her thyroid function normalized without therapy and TRAb persisted negative.

### Case 2

A 23-year-old woman was diagnosed with highly active relapsing-remitting MS and was treated with alemtuzumab as first-line therapy. She was an active smoker with no family history of thyroid disease and she had normal thyroid function with undetectable TRAb (<0.1 IU/L, negative if <0.55).

Nine months after the first dose of alemtuzumab, she developed hypothyroidism with borderline TRAb: TSH 13.8 mIU/L, FT4 0.72 ng/dL (nrr 0.71-1.30), TRAb 1.22 IU/L (negative if <1.22). In the following weeks her hypothyroidism became overt and her TRAb became frankly positive: TSH 92.3 mIU/L, FT4 0.18 ng/dL (nrr 0.62-1.32), TRAb 12.7 IU/L (negative if <0.55), TPOAb 847 IU/ml (negative if <35), TgAb 64 IU/ml (negative if <64). Her thyroid ultrasound showed a small dishomogeneous markedly hypoechoic gland of total 4.5 ml volume (normal 9 ± 3 ml), with no nodules. She was started on replacement therapy with LT4–107 mcg daily, reaching a good biochemical control in the following months despite persistently elevated TRAb (31.1 IU/L, negative if <1.22).

At age 25 she became pregnant. At GW 4 her thyroid function was well controlled (TSH 3.4 mIU/L, FT4 0.96 ng/dL (nrr 0.71-1.30)), whereas at GW 8, despite an increase in LT4 dose to 114 mcg daily, the patient was hypothyroid: TSH 8.9 mIU/L. The dose was further increased to 137 mcg daily, with good control of thyroid function: TSH 1.55 mIU/L at GW 16. At week 22 her TRAb were significantly reduced (4.47 IU/L, negative if <1.22) and her TSH was 0.3 mIU/L, thus the LT4 dose was reduced to 125 mcg daily and her thyroid function remained stably controlled until the end of pregnancy. She had a vaginal induced delivery at 41 GW and the baby was healthy. The LT4 dose was maintained 125 mcg daily and the last follow-up visit 8 weeks post-partum showed optimal control of her thyroid function: TSH 1.24 mIU/L.

### Case 3

A woman with no previous history of thyroid disorders was diagnosed with MS at the age of 28 and was treated with steroids, glatiramer acetate (an immunomodulator that reduces relapse rates by switching from an inflammatory to an anti-inflammatory state), dimethyl fumarate (a nrf2 pathway activator, that exerts anti-inflammatory and cytoprotective effects) (17) and two doses of alemtuzumab one year apart. About two years after the first administration, her routine blood tests revealed mild hyperthyroidism with TSH 0.14 mIU/L, FT4 1.1 ng/dL (nrr 0.9-1.8), FT3 5.1 pg/mL (nrr 2.3-4.2), positive TRAb (75 IU/L, ref. range <0.55) and negative TPOAb and TgAb. In the following month her thyroid function switched to overt hypothyroidism (TSH 60 mIU/L, FT4 0.48 ng/dL (nrr 0.89–1.76)) and she was started on LT4 replacement therapy 75 mcg daily, with poor biochemical control despite increasing the dose up to 160 mcg daily (2.2 mcg/kg), switching to a liquid formulation, and after excluding potential causes of gastrointestinal malabsorption. Her TRAb levels reached values >400 IU/L (negative if <0.55) and the measurement of blocking and stimulating TRAb demonstrated the presence of blocking autoantibodies (37%, negative if<20%), but no stimulating antibodies (SI = 0.82, negative if <1.5). A block-and-replace regimen with LT4–125 mcg daily plus propylthiouracil (PTU) 200mg daily was therefore introduced, which finally allowed satisfactory control of her thyroid function and a progressive decrease in TRAb levels. Her thyroid ultrasound showed a small dishomogeneous hypoechoic gland of total 4 mL volume, with no nodules.

At age 33, after one year of block and replace therapy, the patient became pregnant. In the first trimester, a regimen of LT4–137 mcg daily plus PTU 100 mg daily ensured excellent control of her thyroid function: TSH 1.3 mIU/L, FT4 1.34 ng/dL (nrr 0.89-1.76), FT3 2.7 pg/mL (nrr 2.3-4.2). From the second trimester PTU was switched to MMI 2.5 mg daily and the patient remained euthyroid under block and replace treatment, that was shifted to LT4 only therapy at GW 30. During pregnancy her TRAb serum concentrations progressively decreased to 6.4 IU/L (negative if <1.75). At GW 35 she delivered by emergency caesarean section because of pre-eclampsia progressing to HELLP syndrome (Hemolysis, Elevated Liver enzymes, and Low Platelet count); the baby was born preterm but healthy.

After delivery she was kept on LT4 only therapy 125 mcg daily, but after two months her TRAb raised to 11.9 IU/L (negative if <1.75) and overt hyperthyroidism relapsed with TSH <0.008 mIU/L, FT4 1.95 ng/dL (nrr 0.89–1.76), FT3 7.6 pg/mL (ref. range 2.3–4.2), thus she was switched again to block and replace regimen with MMI 20 mg and LT4–125 mcg daily. Ten months after delivery both drugs were discontinued and she underwent radioiodine therapy with steroid prophylaxis for thyroid eye disease (four weekly intravenous methylprednisolone administrations: two doses of 500 mg followed by two doses of 250 mg) (18). Subsequently, the patient developed iatrogenic hypothyroidism and was started on LT4 replacement therapy, with suboptimal control of thyroid function due to fluctuating TSH from 0.36 mIU/L to 8.4 mIU/L, normal concentrations of FT4 and FT3 and persistence of high TRAb levels: 37.8 IU/L (negative if <1.75).

Seven months after radioiodine therapy, she developed moderate inactive unilateral Graves’ orbitopathy in the right eye, characterized by progressive proptosis and orbital pain (19). Orbital magnetic resonance imaging scan confirmed the presence of exophthalmos and enlargement of the extraocular muscles of the right eye. Eight months after disease onset she showed a clinical activity score (CAS) of 4 out of 10 points with oppressive and gaze evoked orbital pain, increase in proptosis and reduction of ocular motility, thus she was started on therapy with selenium 200 mcg daily (19–21). Two months later her orbital disease had inactivated (CAS 0/10) and persisted stable at the end of follow-up, two years after radioiodine treatment.

### Case 4

A 21-year-old woman was diagnosed with MS and treated with steroids, interferon and fingolimod. At the age of 36 she was treated with one dose of alemtuzumab and after eleven months she developed GD with overt hyperthyroidism: TSH <0.005 mIU/L, FT4 6.6 ng/dL (nrr 0.9–1.7), TRAb 30 IU/L (negative if <0.55), positive TPOAb and TgAb. Her thyroid ultrasound showed a dishomogeneous hypoechoic gland with normal volume (12 ml), increased vascularity and some sub-centimetric cystic nodules. She was started on MMI therapy and after six months she was switched to block-and-replace regimen because of inadequate disease control. The second dose of alemtuzumab was deferred and carried out two years after the first infusion. An attempt of thyroid drugs discontinuation was made, followed by relapse of hyperthyroidism after one year, when MMI alone was re-started. The patient came to our attention at the age of 40, with good control of hyperthyroidism under MMI 5 mg daily: TSH 0.16 mIU/L, FT4 1.41 ng/dL (nrr 0.93–1.70), FT3 3.34 pg/mL (nrr 2-4.4), TRAb 10.5 IU/L (negative if <1.75). She expressed the desire to immediately seek pregnancy due to her age and good temporary control of her MS disease, thus she was switched to PTU 100 mg daily, followed by good control of thyroid function.

At age 41 she became pregnant with successful assisted reproductive technology and heterologous fertilization. During pregnancy her PTU dose was tapered to 75 mg daily and switched to MMI 2.5 mg daily from the second trimester onwards, with stable control of thyroid function and progressive decrease of serum TRAb concentrations. At the end of the third trimester, she had TSH 0.67 mIU/L, FT4 0.96 ng/dL (nrr 0.93–1.70) and TRAb 2.35 IU/L (negative if <1.75).

Delivery occurred at term by planned caesarean section at GW 40 and the baby was healthy. In the postpartum period, good thyroid control was maintained with low-dose MMI (1.25 mg daily) long-term, and her TRAb concentrations remained stably low.

Two years after delivery the biochemical control deteriorated again with TSH <0.005 mIU/L, FT4 2.13 ng/dL (nrr 0.93–1.70), FT3 6.32 pg/mL (nrr 2-4.4) and TRAb 5.12 IU/L (negative if <1.75), thus her MMI was increased to 5 mg daily and she underwent radioiodine therapy with steroid prophylaxis for thyroid eye disease. After six months she developed mild hypothyroidism (TSH 0.014 mIU/L; FT4 0.83 ng/dL (nrr 0.89-1.90), FT3 2.99 pg/mL (nrr 1.82-4.62), and was started on replacement therapy with LT4–25 mcg daily. At the last follow-up visit she had TSH 8.38 mIU/L, FT4 0.65 ng/dL (nrr 0.93–1.7) and FT3 2.0 pg/mL (nrr 2-4.4) and the LT4 dose was increased to 50 mcg daily.

## Literature review

### Characteristics of patients with alemtuzumab-induced GD or TRAb-positive hypothyroidism in pregnancy

In addition to the four cases described in this case-series, seven other cases of pregnant women with alemtuzumab-induced GD or TRAb-positive hypothyroidism have been reported in the scientific literature, for a total of eleven cases to date (**Table 1**). Prior to alemtuzumab, some of these patients had received other therapies for MS, including steroids, interferon, fingolimod, dimethyl fumarate and glatiramer acetate. The mean ± SD age at pregnancy start was 32.6 ± 5.0 years, with a median thyroid dysfunction time onset of 22 months (IQR 15–32 months) after the first alemtuzumab infusion. None of the patients had a history of thyroid disease prior to treatment. In seven cases thyroid dysfunction developed during pregnancy: four of them during the first trimester, one in the second and two in the third, respectively. In the remaining four patients, thyroid dysfunction had developed between 12 and 49 months before pregnancy. The predominant thyroid dysfunction was hyperthyroidism due to GD in seven patients, and TRAb-positive hypothyroidism in the other four, with two of them presenting spontaneous shifts between the two thyroid conditions (8, 13, 22–26).

**Table 1:** Clinical characteristics, therapeutic management and maternal-neonatal outcomes of described cases of alemtuzumab-induced Graves’ disease and TRAb-positive hypothyroidism.

	Age at conception (years)	Age at MS diagnosis (years)	History of thyroid diseases before ALZ	Previous MS DMT	Number of ALZ doses before thyroid dysfunction onset	Time from first ALZ dose to thyroid diagnosis (months)	Onset of thyroid dysfunction during pregnancy	Time from thyroid diagnosis to pregnancy (months)	Predominant thyroid dysfunction during pregnancy	Thyroid therapy during pregnancy	TRAb at thyroid dysfunction diagnosis: IU/L (positive threshold)	TRAb in the third trimester of pregnancy: IU/L (positive threshold)	Delivery	Newborn
Case 1 (described above)	29	19	No	IFN + fingolimod	2	29	Yes (third trimester)	–	Hyperthyroidism	MMI	9.2 (<1.8)	3.8 (<1.8)	Scheduled caesarean section at GW 37	Healthy
Case 2 (described above)	25	23	No	None	1	11	No (before pregnancy)	12	Hypothyroidism	LT4	12.7 (<0.55)	4.47 (<1.22)	Induced vaginal delivery at GW 41	Healthy
Case 3 (described above)	33	28	No	glatiramer + dimetilfumarate	2	22	No (before pregnancy)	30	Hypothyroidism	B&R (LT4 + PTU/MMI)	74.7 (<0.55)	6.4 (<1.75)	Emergency cesarean section at GW 35 for pre-eclampsia progressing to HELLP syndrome	Preterm but healthy
Case 4 (described above)	41	21	No	IFN + fingolimod	1	11	No (before pregnancy)	49	Hyperthyroidism	PTU/MMI	30 (<0.55)	2.35 (<1.75)	Scheduled caesarean section at GW 40	Healthy
Case 5 (13)	36	29	Not reported	IFN + fingolimod	3	36	No (before pregnancy)	36	Hyperthyroidism	PTU/CBZ	13 (<1.8)	128 (<1.8)	Spontaneous delivery at GW 40, vacuum-assisted due to risk of asphyxia. No complications occurred	High TRAb (240 IU/L). On day 10 onset of hyperthyroidism, treated with ATD and beta-blockers.
Case 6 (22)	31	Not reported	Not reported	Not reported	Not reported	32	Yes (first trimester)	–	Hyperthyroidism	PTU/CBZ	2.94 (< 0.1).	Not reported	Spontaneous delivery at GW 40	Healthy
Case 7 (23)	28	Not reported	No	Not reported	Not reported	26	Yes (second trimester)	–	Hypothyroidism	LT4	Positive (unspecified)	Not reported	At term	On day 5 normal TSH with elevated FT4. No ATD needed due to spontaneous progressive normalization of thyroid function.
Case 8 (23)	37	Not reported	No	Not reported	Not reported	19	Yes (first trimester)	–	Hypothyroidism	LT4	Elevated (unspecified)	Not reported	At term	On day 1 normal FT4 with elevated TSH. No ATD needed due to spontaneous progressive normalization of thyroid function.
Case 9 (24)	36	Not reported	No	Not reported	Not reported	15	Yes (first trimester)	–	Hyperthyroidism	CBZ	15.2 (<1.5)	40 (<1.5)	Spontaneous delivery at GW 38	Transient hyperthyroidism at birth (FT4.100 pmol/L; TSH,0.01 mIU/L) requiring ATD.
Case 10 (25)	27	Not reported	No	Not reported	Not reported	22	Yes (third trimester)	–	Hyperthyroidism	PTU	40 (<1.0)	Not reported	Delivery at GW 35	Hyperthyroidism day 3: TSH 0.03 mIU/L; FT4 35.1 pmol/L (RR 10.0 to 19.8 pmol/L); FT3 23.1 pmol/L (RR 3.5 to 6.5 pmol/L); TRAB.40 IU/L (<1.0). CBZ required until age 3 months.
Case 11 (26)	36	27	No	Not reported	3	108	Yes (first trimester)	–	Hyperthyroidism	MMI	20.6 (<1.75)	35.3 (<1.75)	Delivery at GW 41	On day 1 euthyroid status with high TRAb, on day 10 onset of hyperthyroidism No ATD needed due to progressive normalization of the thyroid function.

ALZ, alemtuzumab; B&R, block and replace; CBZ, carbimazole; DMT, disease-modifying therapy; FT3, free tri-iodothyronine; FT4, free thyroxine; GW, gestational week; IFN, interferon; LT4, levothyroxine; MMI, methimazole; MS, multiple sclerosis; PTU, propylthiouracil; TRAb, anti-thyroid-stimulating hormone receptor antibodies; TSH, thyroid-stimulating hormone.

### Clinical management and outcomes of alemtuzumab-induced GD or TRAb-positive hypothyroidism in pregnancy

Considering the seven total hyperthyroid cases, the two patients presented in this case series (Cases 1 and 4) achieved good control of thyroid function during pregnancy with ATD therapy. Among the published cases, one patient (Case 5) required a progressive increase of ATD dosage during the first 20 GWs, after which thyroid function normalized despite persistently elevated TRAb. Another woman (Case 6) was treated with PTU until the end of the first trimester when it was discontinued, later re-started at GW 18 because of relapse of hyperthyroidism and definitively withdrawn at GW 25 for remission of hyperthyroidism with negative TRAb (0.859 IU/L, negative if < 0.1). In two other cases (Cases 9 and 11) ATD dose adjustments were required due to exacerbation of hyperthyroidism during the pregnancy course. Case 10 required high doses of ATD until the end of pregnancy to maintain a good control of her thyroid function.

Among the four patients with TRAb-positive hypothyroidism, three were treated with LT4 alone (Cases 2, 7 and 8), obtaining optimal control of thyroid function except for Case 2, with uncorrected subclinical hypothyroidism in the first trimester and subsequent recover in the following weeks until delivery. In particular, cases 7 and 8 achieved normalization of thyroid function with LT4 treatment alone despite severe hypothyroidism at onset: TSH >100 mIU/L, FT4 3.7 pmol/L (nrr 9–15 pmol/L) at GW 17 and TSH 47.9 mIU/L, FT4 8.8 pmol/L (nrr 10–16 pmol/L) at GW 5, respectively. No data about long-term neurocognitive outcomes of newborns are available. In Case 3 the control of thyroid function was maintained excellent throughout pregnancy with a block-and-replace regimen. Such treatment option was preferred due to poor control of thyroid function on LT4-only therapy, and high TRAb titer.

In the majority of cases, serum TRAb titers were measured during the second or third trimester and close fetal monitoring by ultrasound was performed to assess growth and appearance of signs of thyrotoxicosis, such as goitre or tachycardia. As reported in **Table 1**, six newborns showed thyroid function alterations at birth or in the first post-partum days (Cases 5, 7, 8, 9, 10 and 11), with ATD therapy required in Cases 5, 9 and 10. In particular, the newborn of Case 5 was closely monitored in the intensive care unit for elevated risk of perinatal hyperthyroidism due to very high TRAb titer (240 IU/L). On day 10, the newborn developed signs of overt hyperthyroidism (weight gain cessation and heart rate increase) with FT4 47.9 pmol/L (nrr 8–21 pmol/L) and FT3 16.7 pmol/L (2.8–7 pmol/L). Treatment with carbimazole (CBZ; 0.5 mg/kg daily) and propranolol (2 mg/kg daily) was started and gradually tapered, with normalization of symptoms and thyroid function after three weeks, followed by drug discontinuation after 6 weeks, when also TRAb titers had become negative. In case 9 ATD were required considering overt hyperthyroidism (FT4–100 pmol/L; TSH 0.01 mIU/L). Similarly, in Case 10 the newborn presented with overt hyperthyroidism and very high TRAb titer at day 3 (TSH 0.03 mIU/L; FT4 35.1 pmol/L (RR 10.0 to 19.8 pmol/L); FT3 23.1 pmol/L (RR 3.5 to 6.5 pmol/L); TRAB.40 IU/L (<1.0)), requiring CBZ treatment until age 3 months.

In Case 7 the newborn presented normal TSH with elevated FT4 31.5 pmol/L (nrr 12-22) on day 5, while in Case 8 the baby showed normal FT4 with elevated TSH (13.02 mIU/L) on day 1; both normalized spontaneously without any treatment by day 11.

In Case 11 the newborn was hyperthyroid with high TRAb titers: TSH 0.048 mIU/L, FT4 28.84 pg/ml (nrr 8-19), FT3 8.65 pg/ml (nrr 1.8-4.2), TRAb 28.2 IU/L (negative if <0.55 IU/L). No ATD therapy was started owing the rapid progressive spontaneous normalization of FT4–15 days after delivery (TSH 0.037 mIU/L, FT4 14.98 pg/ml (nrr 8-19)), followed by TRAb negativization 75 days after delivery: TRAb 0.45 IU/L (negative if <0.55 IU/L), with TSH 3.269 mIU/L, FT4 13.31 pg/ml (nrr 8-19).

## Discussion

This case series provides important clinical insights into the management of alemtuzumab-induced thyroid dysfunction during pregnancy. The current 2019 ETA guidelines recommend that women who are pregnant or are seeking pregnancy, within four years from the last alemtuzumab dose, shall undergo monthly thyroid function testing rather than every three months (11). Based on our experience, we believe that such recommendation is particularly appropriate.

In this case series of eleven pregnant women with alemtuzumab-induced thyroid dysfunction, the disease course was notably variable, in line with previous findings. Compared with the spontaneous form, alemtuzumab-induced GD frequently shows fluctuations of thyroid function between states of hypothyroidism and hyperthyroidism, not simply due to overtreatment or poor compliance (6, 8–10). The pathophysiological mechanism underlying the fluctuating thyroid function is the frequent coexistence of TRAb with stimulating and blocking activity (16). In Case 3 we specifically documented the presence of blocking TRAb, which resulted in poor control of hypothyroidism despite therapy with high-dose LT4. This observation is consistent with previous studies demonstrating that blocking TRAb are responsible for around 40% of post-alemtuzumab cases of hypothyroidism, a proportion significantly higher than the 10% observed in spontaneous autoimmune hypothyroidism (8, 16, 27). The goal to obtain a good control of thyroid function becomes particularly mandatory in pregnant women, considering the adverse effects of both hypothyroidism and hyperthyroidism on either pregnancy or fetal outcomes (12, 28, 29).

An interesting aspect of our case series is the fact that in several cases the onset of thyroid dysfunction occurred during pregnancy. Hyperthyroidism, with GD being the predominant etiology, during pregnancy is rare, occurring in 1–3/1000 pregnancies, with a prevalence of 0.1–0.3%. As many autoimmune diseases, GD typically improves or shows a lower clinical incidence throughout the gestational period, due to the partial downregulation of cell-mediated immunity and the enhancement of maternal-fetal tolerance (12, 30, 31). However GD incidence varies during the different phases of pregnancy. A Danish population-based study demonstrated that GD incidence peaked during the first gestational trimester (32). As pregnancy progresses, changes in the immunologic response decrease incidence and lead to symptoms improvement, until the postpartum period, when a rebound of the immune system can lead to GD exacerbation (33). This is not surprising, considering that pregnancy is a condition of complex dynamic immunomodulation, rather than constant immunosuppression as deemed in the past. The first trimester is characterized by a pro-inflammatory state, followed by immune tolerance during the second trimester, and a subsequent re-exacerbation of inflammation at the end of third trimester, essential to trigger labor (34).

During the whole gestational period it is imperative to maintain an optimal control of thyroid function to minimize both maternal and fetal risks. In this case series we described the clinical management of pregnant patients with alemtuzumab-induced autoimmune thyroid dysfunction, challenged by the frequent coexistence of stimulating and blocking TRAb causing relatively abrupt changes in thyroid function, and a consistent risk for fetal/neonatal thyroid dysfunction, due to TRAb positivity often at high titers. ATD is the first treatment of choice for GD during pregnancy, with PTU recommended in the first trimester, due to its lower rate of congenital malformations compared with CBZ/MMI when administered during organogenesis: 3% versus 5%, respectively (12, 35–37). PTU has a higher risk of hepatotoxicity (38), thus it has been usually recommended to change back to CBZ/MMI after GW 16. Recent 2026 guidelines from the American Thyroid Association highlights that the evidence supporting such switch is unclear (12, 37). In cases of fluctuating thyroid function poorly controlled with an ATD-only therapeutic regimen, an alternative option is the block and replace regimen with combined ATD and LT4. However, in the block and replace regimen ATD doses are usually high, while it is essential not to overtreat pregnant women, to avoid negative consequences of fetal and neonatal hypothyroidism and ATD side effects (12). In our case series, the block and replace regimen with low-dose ATD represented a valid therapeutic choice in one pregnant woman with TRAb-positive hypothyroidism (Case 3), since the LT4-only regimen did not ensure a stable control of her thyroid function. Additional evidence in larger patient cohorts is needed before suggesting block and replace therapy as an alternative therapeutic option in TRAb-positive hypothyroidism, especially during pregnancy. Importantly, there are no available neurocognitive long-term outcomes of the newborns of mothers developing overt TRAb-positive hypothyroidism during pregnancy. Differently from Hashimoto’s thyroiditis, TRAb-positive hypothyroidism may have a sudden onset similarly to GD, with potential severe consequences on fetal brain development if not promptly diagnosed and corrected. In case 3 the mother underwent emergency caesarean section at GW 35 because of pre-eclampsia progressing to HELLP syndrome; the baby was born preterm but healthy. This represents a clinically significant obstetric event, although the available data do not establish that it was related to the underlying thyroid disorder, or its treatment, or other causes.

An additional major risk of GD during pregnancy is the transplacental transfer of maternal TRAb antibodies, which exert biological function on the fetal thyroid gland. Fetal or neonatal hyperthyroidism usually occur when maternal serum TRAb concentrations are three times or above the upper reference limit, especially in the third trimester of pregnancy, or in the early post-partum period (12, 14, 39). The optimal management of this condition includes close fetal and neonatal thyroid ultrasound and heart rate monitoring, with regular contacts between endocrinologists and obstetrician, maternal-fetal medicine and neonatal specialists (12). Indeed, in this case series six newborns developed TRAb-mediated neonatal thyroid dysfunction, despite good control of maternal thyroid function. In particular, three newborns developed overt neonatal hyperthyroidism requiring ATD therapy, and other three showed transient neonatal hyperthyroidism or hypothyroidism spontaneously improved. None of the reported cases resulted in significant maternal or fetal complications. These cases underscore the importance of adequate neonatal monitoring even in presence of good thyroid function control during pregnancy, considering the high risks primarily deriving from raised maternal serum TRAb titer.

The main limitations of this study are the small number of reported cases to date, and their observational and heterogeneous nature. Current evidence consists in few individual case reports/series of pregnant women with different timing and phenotypes of alemtuzumab-induced thyroid dysfunction, and heterogeneous treatment strategies, overall clinical management, laboratory assays and reference ranges, and duration of maternal and neonatal follow-up. The principal strengths of this study are represented by the rarity of the subject, the detailed longitudinal characterization of the four institutional cases, their integration with the limited published literature currently available, and the focus on both maternal and fetal/neonatal outcomes. Dedicated studies should be encouraged to increase the quality of available scientific evidence, possibly including also long-term offspring neurocognitive data.

In conclusion, women with thyroid dysfunction induced by alemtuzumab often require complex and challenging clinical management during gestation, due to fluctuating thyroid status, and TRAb-mediated fetal/neonatal risk of thyroid dysfunction. To minimize risks for both pregnancy and fetal outcomes in these patients, close monitoring of thyroid function and frequent treatment adjustments are required to maintain appropriate maternal thyroid hormone levels. These women can experience successful pregnancies with close thyroid function monitoring and personalized therapeutic approaches, to be provided in an appropriate specialist setting. A multidisciplinary team including endocrinologist, obstetrician, maternal-fetal medicine and neonatal specialists is recommended, especially in cases of pregnant women with high serum TRAb titers.
